# Report on the Progress of the Journal Food Technology and Biotechnology

**Published:** 2024-03

**Authors:** Iva Grabarić Andonovski

**Affiliations:** Editor of Food Technology and Biotechnology journal; Faculty of Food Technology and Biotechnology, University of Zagreb, Pierottijeva 6, 10000 Zagreb, Croatia

Journal Food Technology and Biotechnology is steadily on the path of progress, evolving each year in various aspects. Journal’s aims and scope are now defined more precisely, providing authors clear directions to find the appropriate journal for the dissemination of their research results. The website has been upgraded to the newest version of Joomla, and website accessibility features have been installed, in accordance with the web accessibility Directive (EU) 2019/882 (*1*), and HTML functionality for recently published articles is enabled.

Last year, 46 papers were published on 550 pages in four issues, of which four were previously presented at CEFOOD2022 - 11th Central European Congress on Food and Nutrition, held in Čatež ob Sava, Slovenia from 27 September to 1 October 2022, published in issue 3/2023, and six papers were previously presented at the 10th Anniversary International Congress of Food Technologists, Biotechnologists and Nutritionists held in Zagreb from 30 November to 2 December 2022, as a result of the journal collaboration with Slovenian Microbiological Society and Croatian Society of Food Technologists, Biotechnologists and Nutritionists, respectively.

Editorial policy and instructions for authors have been updated, as well as general information and information on indexing. According to the Journal Citation Reports® (*2*), Journal Impact Factor (JIF) for 2023 is 2.4, which positions the journal in Q3 (94/142 journals in food science and technology and 114/158 journals in biotechnology and applied microbiology). However, the 5-year JIF is 4.3, which positions it in Q2. Journal Citation Indicator (JCI) is 0.46. According to the SCImago Journal & Country Rank (*3*), the journal is in Q2 (SJR=0.437), with H-index of 76, and CiteScore of 4.0.

The journal’s Environmental policy has been updated. An option for authors to choose SDGs related to the subject of the paper has been added to the online submission system, logotypes with the link to the SDGs selected for the paper are posted at the journal’s website and an editorial explaining the need to join the UN SDG Publishers Compact Initiative and which steps the journal has taken has been published (*4*). Also, the journal’s Equity, diversity and inclusion policy is being implemented in the editorial work, with the special emphasis on gender equity (*e.g.* 58% female Field editors, 39% male and 3% others were assigned).

Journal workflows have been strengthened and the FTB Comet online submission system is constantly being improved, trying to increase the speed and efficiency of the peer review process. In 2023, the Editorial office received 430 submissions, of which 364 (85%) were rejected, 48 were accepted, 20 manuscripts were withdrawn by the authors, and the rest of them are under evaluation. The main reasons for rejection were the following: (*i*) lack of novelty, (*ii*) the topic was outside the scope of the journal, (*iii*) incoherent presentation of the results, (*iv*) flaws in methodology, including the lack of statistical analysis or inadequate use of methods, (*v*) incomplete product analysis, (*vi*) lack of discussion (no comparison of the results with previously published data). On average, the initial triage took 9 days, the field editors needed 20 days to reach the decision, and the reviewers needed 114 days to complete the reviewing process. Average time to reach a decision about the acceptance or rejection of the submission was 185 days. The main reason why reviewing process takes so long is the reviewers’ fatigue, since in many cases we needed to add 15−20 reviewers to obtain two reviews. Also, multiple revisions were requested from the authors, which prolonged the time to reach the final decision. Manuscripts submitted to the journal were evaluated by 222 field editors and reviewers in total.

Numerous upgrades have been made to implement the API connectivity of the FTB Comet system to iThenticate 2.0, which enables the detection of the work that was plagiarised or produced with generative AI tools (*5*).

We are also introducing viewpoints as a new article category in our journal. For now, we are only publishing invited viewpoints on the topics relevant for the journal scope or journal editing procedures and policies. The first one has been written by our Linguistic editor Zrinka Pongrac Habdija, on the use of AI in scholarly communication, available in this issue (*6*).

As a result of all these activities, the journal was awarded Directory of Open Access Journals Seal (*7*) in November 2023, a recognition of the highest quality and best practices in Open Access publishing. Only 10% of journals indexed in DOAJ have been awarded the Seal, and our journal is currently the only one in Croatia that has received this award.

We are very proud that all the initiatives and hard work put into the journal organization and editing are recognized. This gives us strength to continue our efforts to become one of the leading diamond open access scholarly publications in the fields covered by our journal.
